# Association of various glycemic variability indices and vascular outcomes in type-2 diabetes patients: A retrospective study

**DOI:** 10.1097/MD.0000000000010860

**Published:** 2018-05-25

**Authors:** Lei Tong, Chen Chi, Zhiguo Zhang

**Affiliations:** a Shanghai Institute of Endocrine and Metabolic Diseases, Shanghai Clinical Center for Endocrine and Metabolic Diseases, State Key Laboratory of Medical, Economics, Ruijin Hospital, Shanghai Jiao Tong University School of Medicine; b Department of Cardiology, Shanghai Tenth People's Hospital, Tongji University School of Medicine, Shanghai, China.

**Keywords:** diabetes, glycemic variability, self-monitored blood glucose, vascular outcomes

## Abstract

Both blood glucose (BG) level and glycemic variability (GV) significantly associate with diabetes-related complications. However, the criterion standard in GV assessment is absent. We aimed to compare different GV indices in association of vascular outcomes.

Ten commonly used GV indices based on self-monitored BG data were calculated, and their associations of vascular outcomes including coronary artery disease (CAD), stroke, and chronic kidney disease (CKD) were compared.

In total, 288 type 2 diabetes patients (66.5 ± 11.1 years old) were included in present analysis. Spearman correlation analysis showed that only mean amplitude of glycemic excursions (MAGE) significantly correlated with both estimated glomerular filtration rate and urinary albumin creatinine ratio (*P *≤ .03). In Cochran-Armitage trend test, vascular outcomes were significantly associated with the increment of BG risk index and MAGE (*P *≤ .03). After adjustment for potential confounders, multiple logistic regression results suggested that BG risk index and MAGE still significantly associated with these three vascular outcomes (*P *≤ .01), whereas the other GV indices did not. Receiver operating characteristic curve analysis showed that the abilities of BG risk index and MAGE were similar in identifying CAD, stroke, or CKD.

BG risk index and MAGE were better associated with vascular outcomes than other GV indices in type 2 diabetes patients.

## 1 Introduction

Diabetes mellitus (DM), especially type-2 DM (T2DM) which accounts for more than 90% of all DM cases, is a world-wide healthy concern. The poor-controlled blood glucose (BG) significantly increases the risk of DM-related vascular complications including coronary artery disease (CAD), stroke, kidney disease, and so on.^[1]^ The role of glycemic level in DM-related complications has been well-established, and emerging clinical data show that, not only glycemic level but also its variability (glycemic variability, GV) are both independent risk factors of cardiovascular events.^[2]^ However, the recommendations of current guideline of DM management is based on glycemic level, and the level of glycated hemoglobin A1c (HbA1c) has become the *de facto* criterion standard throughout the management of DM.^[1]^ This glycemic level–based treatment strategy would ignore the role of GV in the progression of DM-related vascular complications.

The application of GV in clinical practice meets with some problems. The first was about the calculation of GV indices. A dozen mathematical approaches were developed during past decades to assess GV. Each method has its own characteristics, but till now, none of them is considered as a criterion standard in GV assessment.^[3]^ With the absence of a widely accepted GV parameter, normally a series of GV parameters were calculated in literature, which made GV was too complex to be.^[4]^ Second, some GV indices were specially designed for continuous glucose monitoring (CGM) data. CGM provides more information about GV than self-monitored blood glucose (SMBG) data.^[3]^ SMBG were still commonly used in routine clinical practice because it was convenient and cost-effective. Moreover, unlike CGM data, SMBG-based GV indices could be calculated without specific software. Thus, we investigated and compared the SMBG-based GV indices in association with vascular endpoints, aiming to find an appropriate method in GV assessment.

## 2 Methods

### 2.1 Characteristics of patients

Consecutive outpatients from June 2016 to May 2017 in Department of Endocrinology, Shanghai Tenth People's Hospital were screened and patients were included in this study if they met all of the following inclusion criteria when they visited the clinics: type 2 diabetes patients (≥18 years old); on treatment with oral hypoglycemic agents and/or insulin and without any changes for at least 1 month; with available data of BG level in 2 consecutive days in the last 2 weeks; fingertip blood samples were tested for BG level 7 times daily; and HbA1c and creatinine level were tested in this visit. The tests were performed by the laboratory in our hospital. This was a retrospective study and the ethical approval was not necessary.

### 2.2 Glycemic variability indices

Before-meal BG (3 times daily), 2-hour postprandial BG (3 times daily), and before-bedtime BG (1 time daily) were measured and recorded. Generally, blood sample were obtained and tested at 6, 9, and 11 am and 1, 5, 7, and 9 pm daily. Mean BG and 10 commonly used GV indices whose calculation could be based on SMBG were calculated, including standard deviation (SD), coefficient of variation (CV), M-index, J-index, area under the curve (AUC), hyperglycemic index, BG risk index, mean of daily differences (MODD), glycemic risk assessment diabetes equation (GRADE), and mean amplitude of glycemic excursions (MAGE).^[5–12]^ Their brief description and formulas of these indices are listed in Table 1. It should be pointed out that although MAGE could be calculated based on SMBG data, it was more suitable for CGM systems (CGMS) than SMBG files and it was very popular among studies with CGMS data. Because of its extensive use in literature, it was included in our present analysis. BG records of the first 2 days were used to calculate MODD when BG were recorded for more than 2 days.

**Table 1:**
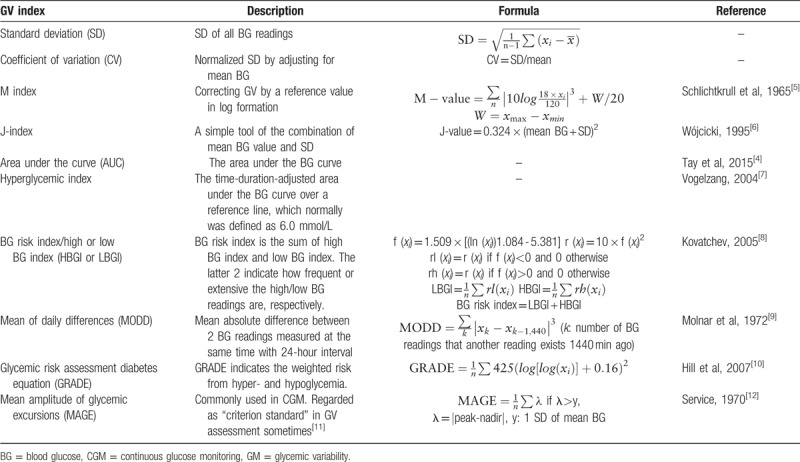
Glycemic variability assessment tools based on self-monitored blood glucose data.

### 2.3 Vascular outcomes assessment

Vascular outcomes, including CAD, stroke, and chronic kidney disease (CKD), were recorded. CAD was defined as previously diagnosed myocardial infarction or stable/unstable angina. Stroke was defined as previously diagnosed ischemic and/or hemorrhagic stroke, and CKD was defined as estimated glomerular filtration rate (eGFR) less than 60 mL/min/1.73 m^2^. The eGFR were calculated with the modified Chinese formula.^[13]^ Urina sanguinis samples were gathered to test the urinary albumin and creatinine level, respectively, and their ratio was calculated (urinary albumin creatinine ratio, UACR).

### 2.4 Statistical analysis

Unless specified, continuous variables were expressed as mean ± SD and categorical variables were expressed as absolute numbers and percentage. Spearman correlation analyses were performed to test the association between kidney function and GV indices. For each GV index, the patients were divided into 3 groups based on tertiles, namely low GV group, medium GV group, and high GV group. The Cochran-Armitage test for trend was performed to assess the tendency. Full-model logistic regression was performed to assess the risk of vascular outcomes in relation to 1-SD change of each GV index after adjustment for age, sex, body mass index, smoking, hypertension, use of insulin, and HbA1c level (mean BG level was not included because of the high collinearity between HbA1c and mean BG level). Finally, to compare the discriminating ability of vascular outcomes of the indices, the receiver operating characteristic (ROC) curve were performed and AUC were compared by the c-test. *P* value <.05 (2-tailed) was considered statistical significant. All statistical analyses were performed with statistical software SAS university edition (SAS Institute, Cary, NC).

## 3 Results

### 3.1 Characteristics

A total of 288 patients were finally included in our present analysis. Characteristics of this population together with the use of antidiabetic agents were summarized in Table 2. The mean age was 66.5 ± 11.1 years. Among these patients, there were 130 (45%) men, 53 (18%) smokers, and 166 (58%) hypertensive patients. Forty-three (15%) of them were with CAD. Seventy-two (25%) of them suffered previously stroke, and 68 (24%) of them were patients with CKD, with the mean eGFR of 62 mL/min/1.73 m^2^.

**Table 2:**
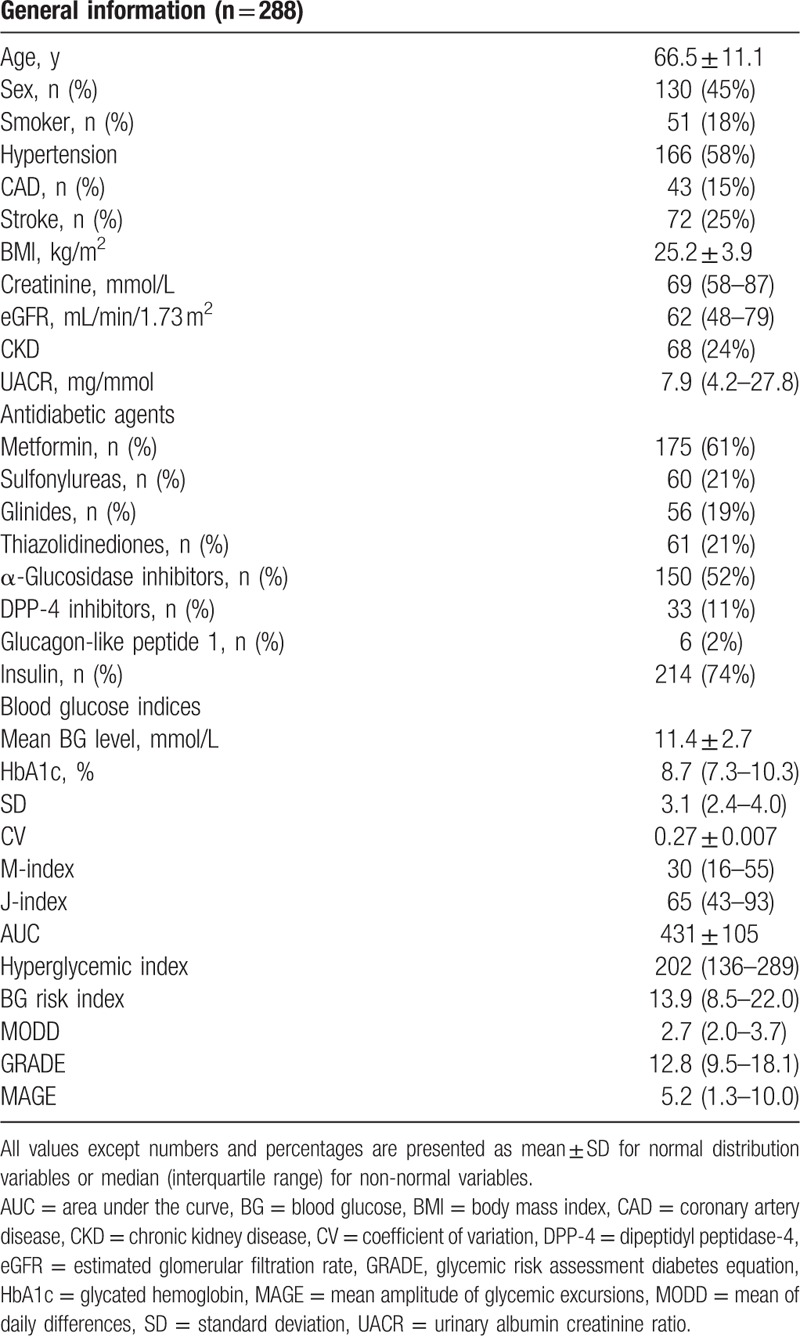
Population characteristics.

### 3.2 Spearman correlation analyses of kidney function and glycemic variability indices

Spearman correlation analyses were performed to test the association of kidney function, which were evaluated by eGFR and UACR, and GV indices. As shown in Table 3, M-index, J-index, AUC, hyperglycemic index, BG risk index, GRADE, and MAGE significantly associated with UACR (*P *≤ .004). The association of other GV indices (SD, CV, and MODD) and UACR did not achieve statistical significance (*P *≥ .09). As for the association between GV indices and eGFR, only MAGE significantly associated with eGFR (*P* = .03), whereas eGFR was not significantly associated with other GV indices (*P *≥ .09).

**Table 3:**
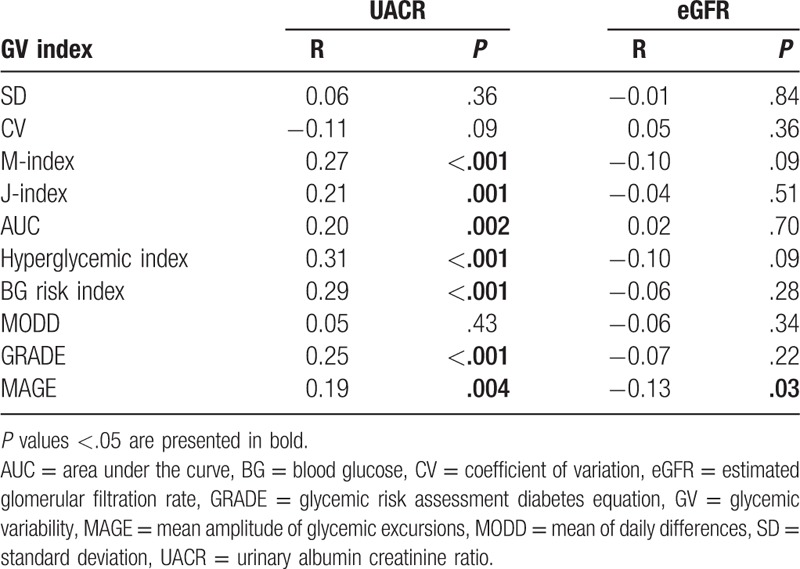
Spearman correlation analysis of glycemic variability indices and continuous kidney function parameters.

### 3.3 Cochran-Armitage trend test

Patients were divided into 3 groups by tertiles, namely low, medium, or high GV group. As shown in Table 4, in trend test, all 3 vascular outcomes including CAD, stroke, and CKD were significantly associated with the increment of BG risk index and MAGE (*P *≤ .03). CAD and stroke, but not CKD, were associated with hyperglycemic index and M-index (*P *≤ .01). These 3 vascular outcomes were not associated with other GV indices in trend test (*P *≥ .06).

**Table 4:**
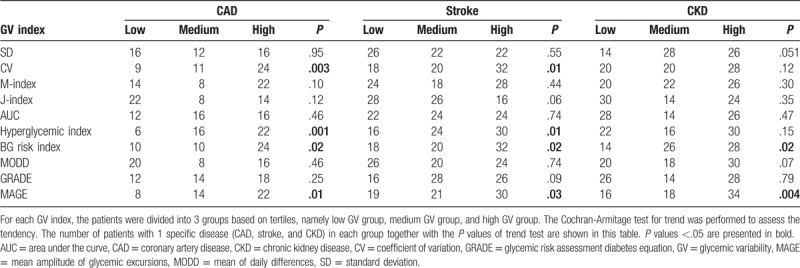
Cochran-Armitage test for trend.

### 3.4 Multiple logistic regression of vascular outcomes and glycemic variability indices

As shown in Table 5, full-model logistic regression was performed to assess the independent association between vascular outcomes and 1-SD change of each GV index after adjustment for potential confounders including age, sex, body mass index, smoking, hypertension, use of insulin, and HbA1c level. Four GV indices were analyzed according to the results of correlation and trend test. After adjustment, BG risk index and MAGE still significantly associated with all 3 vascular outcomes (*P *≤ .01), whereas the association between vascular outcomes and M-index or hyperglycemic index did not remain (*P *≥ .26).

**Table 5:**
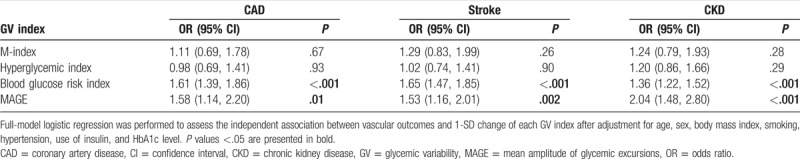
Multiple logistic regression of GV indices and cardiovascular events.

### 3.5 Receiver operating characteristic curve

**Figure 1:**
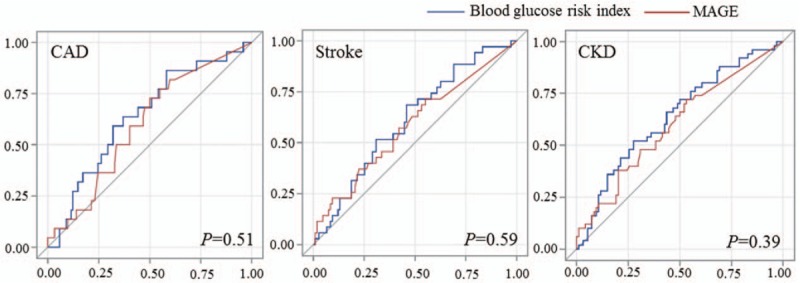
Receiver operating characteristic (ROC) curve. ROC curve was analyzed and c-test was used to compare the area under the curve of blood glucose risk index and MAGE in identifying CAD, stroke, and CKD. CAD = coronary artery disease, CKD = chronic kidney disease, MAGE = mean amplitude of glycemic excursions.

To directly compare the discriminating ability of BG risk index and MAGE, the ROC curve was analyzed and c-test was used to compare the AUC (Fig. 1). The results showed that the abilities of BG risk index and MAGE were similar in identifying no matter CAD, stroke, or CKD (*P *≥ .39).

## 4 Discussion

Many mathematical methods were created for GV assessment, but currently none of them was considered as a criterion standard. Our study showed that, GV independently associated with vascular outcomes after adjustment for potential confounders. Among these GV indices, BG risk index and MAGE were better associated with vascular outcomes than other GV indices.

Various studies have proved that dysglycemia contributed a lot to the development of DM-related complications. Impaired BG regulation leads to not only hyperglycemia, but also increased BG swings compared with healthy subjects.^[3]^ In vitro studies have showed that compared with constant high BG level, high GV could be more harmful to endothelial cells,^[14]^ even with lower mean BG level.^[15]^ Emerging in vivo studies confirmed the independent effects of GV in predicting cardiovascular events.^[16]^ These evidences suggests that, apart from mean BG level, GV also has its important role in accelerating DM-related complications, and GV should be taken into account in daily DM management.^[17]^

With the growing understanding of GV, several mathematical methods were developed to quantitatively assess intraday and interday GV. However, it was a long process to develop an objective, reliable, and sensible GV index. Some indices are easy to calculate, for example, SD and CV, but they seem to be not adequate to evaluate glycemic oscillations. Some methods give enough considerations on different types of glucose fluctuations. For example, BG risk index and MAGE, which take both hyper- and hypo-glycemia into consideration, are able to weight the different contributions of high or low BG level to GV. This might partly explain the reason why BG risk index and MAGE better associate with vascular diseases. However, they seem to be too complicated to apply in routine clinical practice.^[18]^ In addition, with the development of CGM system, more GV assessment tools based on CGM data like continuous overlapping net glycemic action emerged,^[19]^ leading to the chaos of current GV assessment. To date, there is no commonly accepted method to assess GV. With the absence of criterion standard, a series of GV indices would be calculated in literature, and this complicated work made the application of GV assessment in clinical practice more difficult. Our study suggested that, BG risk index and MAGE might be of some advantages because of the adjustment for the skewness of glycemia level in their formulas. Hopefully, this result would simplify the use of different GV indices in the future.

Several clinical issues should be pointed out before the application of GV assessment. First, though more and more data suggested that GV is very important, the role of GV is still in debate, which may be partly owing to the ascertainment of GV assessment tools. In addition, our ROC curve analyses showed that, none of the 3 vascular diseases could be significantly identified by the use of GV indices alone. We hold the opinion that GV will serve as an important complement to glycemic level in diabetes management. Second, optimum duration for adequate GV assessment is not certain. Not only short-term GV, but also long-term GV, were correlated with CV events in literature.^[18]^ On the one hand, the longer assessment is performed, the more details are provided. On the other hand, longer assessment duration might bring more problems to patients, for example, the difficulty of adherence. Third, the cost-effective value should also be taken into consideration. GV assessment, especially CGMS, will cost more money than conventional measures of BG control like HbA1c alone.^[20]^ Fourth, since clinical studies about GV are scare, the normal range and the treatment target of GV indices should be clarified in the future.

There are some limitations in this study. First, as a cross-sectional study, it is very hard to tell the causal relationship between GV and vascular outcomes. High GV might be the risk factor of vascular events. However, patients with different diseases might have different attitudes toward diabetes management. Second, we only have the data about large vascular events, so we had no idea on the association between microvascular changes and GV. Third, our results were built on SMBG data, more studies based on CGM are warranted.

## 5 Conclusion

Our study showed that vascular outcomes were independently associated with GV. The association of BG risk index/MAGE and vascular outcomes were better than other GV indices. This could be taken into consideration in future GV assessment and large clinical trials aiming at GV are warranted.

## Author contributions

**Conceptualization:** Lei Tong.

**Data curation:** Chen Chi.

**Formal analysis:** Chen Chi.

**Methodology:** Lei Tong, Zhiguo Zhang.

**Validation:** Zhiguo Zhang.

**Writing – original draft:** Lei Tong, Chen Chi.

**Writing – review and editing:** Zhiguo Zhang.
